# Supplementary material for: From sequence to enzyme mechanism using multi-label machine learning
Source: BMC Bioinformatics. 2014 May 19;15:150. doi: 10.1186/1471-2105-15-150 (PMC4229970; doi:10.1186/1471-2105-15-150)
Supplement: Additional file 2 — Java code of ml2db. Additional file ml2db_code.tar.gz contains the Java source code to run the multi-label machine learning experiments and save the results to database. The code’s Javadoc is included. [file 1471-2105-15-150-S2.zip › additional file 2/ml2db/utils/doc/uk/ac/standrews/utils/main/database/SqlUtils.html]

SqlUtils


JavaScript is disabled on your browser.


- Overview
- Package
- Class
- Use
- Tree
- Deprecated
- Index
- Help

- Prev Class
- Next Class

- Frames
- No Frames

- All Classes

- Summary:
- Nested |
- Field |
- Constr |
- Method

- Detail:
- Field |
- Constr |
- Method


uk.ac.standrews.utils.main.database

## Class SqlUtils

- java.lang.Object
- - uk.ac.standrews.utils.main.database.SqlUtils

- ---

    

  ```
  public class SqlUtils
  extends java.lang.Object
  ```

  Class

  Version:
  :   29 Jul 2008

  Author:
  :   Luna De Ferrari luna.deferrari-at-ed.ac.uk

- - ### Field Summary

    Fields

    | Modifier and Type | Field and Description |
    | --- | --- |
    | `static int` | `DOUBLE` Basic data type: double (passes without error a parseDouble action) |
    | `static int` | `INTEGER` Basic data type: integer (passes without error a parseInteger action) |
    | `static int` | `MAX_BIGINT_LENGTH` Maximum length in characters for a signed SQL INT BIGINT[(M)] A large integer. |
    | `static int` | `MAX_CHAR_LENGTH` Maximum length in characters for an SQL CHAR column CHAR(M) A fixed-length string. |
    | `static int` | `MAX_INT_LENGTH` Maximum length in characters for a signed SQL INT INT[(M)] A normal-size integer. |
    | `static int` | `NULL` Basic data type: null (the type could not be guessed, eg. |
    | `static int` | `STRING` Basic data type: string (anything that is not an integer or a double...) |
  - ### Constructor Summary

    Constructors

    Constructor and Description

    `SqlUtils()`
  - ### Method Summary

    Methods

    | Modifier and Type | Method and Description |
    | --- | --- |
    | `static java.lang.String` | `getSelectStarSql(java.lang.String table)` Generates a select \* query text (eg. |
    | `static java.lang.String` | `getSelectWhereEqualSql(java.lang.String table, java.lang.String column, java.lang.String equalityConditionValue)` gets sql code for a select on one column of a table, with a where x = y condition specific for that column |
    | `static java.lang.String` | `getSelectWhereEqualSql(java.lang.String table, java.lang.String column1, java.lang.String equalityConditionValue1, java.lang.String column2, java.lang.String equalityConditionValue2)` Select on two columns of a table, with two "where" conditions, , one for each provided column |
    | `static java.lang.String` | `getSelectWhereLikeSql(java.lang.String table, java.lang.String column, java.lang.String equalityConditionValue)` Gets sql code for a select on one column of a table, with a where x like y condition specific for that column |
    | `static java.lang.String` | `getSelectWhereSql(java.lang.String table, java.lang.String column, java.lang.String whereCondition)` gets sql code for a select on one column of a table, with a where condition |
    | `static java.lang.String` | `getSimpleSelectSql(java.lang.String table, java.lang.String column)` gets sql code for a select on one column of a table |
    | `static java.lang.String` | `getSqlForSelectLike(java.lang.String table, java.lang.String column, java.lang.String likeColumn, java.lang.String likeCondition)` Sql code for a select "LIKE" |
    | `static java.lang.String` | `getSqlSelectByIdByRegExp(java.lang.String table, java.lang.String valueColumn, java.lang.String idColumn, java.lang.String id, java.lang.String regExp)` Generates the sql command for a select of a value corresponding to a certain id AND matching a certain regex string |
    | `static java.lang.String` | `getSqlSelectByRegExp(java.lang.String table, java.lang.String column, java.lang.String regExp)` Generates the sql command for a select matching a certain regex string |
    | `static java.lang.String` | `getSqlToDropTable(java.lang.String tableName)` Get sql to drop table |
    | `static java.lang.String` | `surroundWith(java.lang.String text, java.lang.String surrounding)` Adds a surrounding string (usually an apex or similar) as prefix and suffix of the given text. |

    - ### Methods inherited from class java.lang.Object

      `equals, getClass, hashCode, notify, notifyAll, toString, wait, wait, wait`

- - ### Field Detail


    - #### DOUBLE

      ```
      public static final int DOUBLE
      ```

      Basic data type: double (passes without error a parseDouble action)

      See Also:
      :   Constant Field Values


    - #### INTEGER

      ```
      public static final int INTEGER
      ```

      Basic data type: integer (passes without error a parseInteger action)

      See Also:
      :   Constant Field Values


    - #### MAX\_BIGINT\_LENGTH

      ```
      public static final int MAX_BIGINT_LENGTH
      ```

      Maximum length in characters for a signed SQL INT BIGINT[(M)] A large
      integer. The signed range is -9223372036854775808 to 9223372036854775807.
      The unsigned range is 0 to 18446744073709551615.
      http://dev.mysql.com/doc/refman/5.1/en/numeric-type-overview.html

      See Also:
      :   Constant Field Values


    - #### MAX\_CHAR\_LENGTH

      ```
      public static final int MAX_CHAR_LENGTH
      ```

      Maximum length in characters for an SQL CHAR column CHAR(M) A
      fixed-length string. The range of M is 0 to 255 characters.

      See Also:
      :   Constant Field Values


    - #### MAX\_INT\_LENGTH

      ```
      public static final int MAX_INT_LENGTH
      ```

      Maximum length in characters for a signed SQL INT INT[(M)] A normal-size
      integer. The signed range is -2147483648 to 2147483647. The unsigned
      range is 0 to 4294967295.
      http://dev.mysql.com/doc/refman/5.1/en/numeric-type-overview.html

      See Also:
      :   Constant Field Values


    - #### NULL

      ```
      public static final int NULL
      ```

      Basic data type: null (the type could not be guessed, eg. the list was
      empty)

      See Also:
      :   Constant Field Values


    - #### STRING

      ```
      public static final int STRING
      ```

      Basic data type: string (anything that is not an integer or a double...)

      See Also:
      :   Constant Field Values
  - ### Constructor Detail


    - #### SqlUtils

      ```
      public SqlUtils()
      ```
  - ### Method Detail


    - #### getSelectStarSql

      ```
      public static java.lang.String getSelectStarSql(java.lang.String table)
      ```

      Generates a select \* query text (eg. SELECT \* FROM mytable )

      Parameters:
      :   `table` -

      Returns:
      :   a string containing the sql query text


    - #### getSelectWhereEqualSql

      ```
      public static java.lang.String getSelectWhereEqualSql(java.lang.String table,
                                            java.lang.String column,
                                            java.lang.String equalityConditionValue)
      ```

      gets sql code for a select on one column of a table, with a where x = y
      condition specific for that column

      Parameters:
      :   `table` - the table to select from
      :   `column` - the column to select from
      :   `equalityConditionValue` - the x value for the: where column = 'x' condition.

      Returns:
      :   sql code for a select on one column of a table, with a where
          condition specific for that column


    - #### getSelectWhereEqualSql

      ```
      public static java.lang.String getSelectWhereEqualSql(java.lang.String table,
                                            java.lang.String column1,
                                            java.lang.String equalityConditionValue1,
                                            java.lang.String column2,
                                            java.lang.String equalityConditionValue2)
      ```

      Select on two columns of a table, with two "where" conditions, , one for
      each provided column

      Parameters:
      :   `table` - the table to select from
      :   `column1` - the column to select from
      :   `equalityConditionValue1` - the x1 value for the: where column1 = 'x1' condition.
      :   `column2` - the column to select from
      :   `equalityConditionValue2` - the x2 value for the: where column2 = 'x2' condition.

      Returns:
      :   SQL for a select on two columns of a table, with two "where"
          conditions, one for each provided column


    - #### getSelectWhereLikeSql

      ```
      public static java.lang.String getSelectWhereLikeSql(java.lang.String table,
                                           java.lang.String column,
                                           java.lang.String equalityConditionValue)
      ```

      Gets sql code for a select on one column of a table, with a where x like
      y condition specific for that column

      Parameters:
      :   `table` - the table to select from
      :   `column` - the column to select from
      :   `similarityConditionValue` - the x value for the: where column like '%x%' condition.

      Returns:
      :   sql code for a select on one column of a table, with a where-like
          condition specific for that column


    - #### getSelectWhereSql

      ```
      public static java.lang.String getSelectWhereSql(java.lang.String table,
                                       java.lang.String column,
                                       java.lang.String whereCondition)
      ```

      gets sql code for a select on one column of a table, with a where
      condition

      Parameters:
      :   `table` - the table to select from
      :   `column` - the column to select from

      Returns:
      :   the sql for a simple one column unique and ordered query


    - #### getSimpleSelectSql

      ```
      public static java.lang.String getSimpleSelectSql(java.lang.String table,
                                        java.lang.String column)
      ```

      gets sql code for a select on one column of a table

      Parameters:
      :   `table` - the table to select from
      :   `column` - the column to select from

      Returns:
      :   the sql for a simple one column unique and ordered query


    - #### getSqlForSelectLike

      ```
      public static java.lang.String getSqlForSelectLike(java.lang.String table,
                                         java.lang.String column,
                                         java.lang.String likeColumn,
                                         java.lang.String likeCondition)
      ```

      Sql code for a select "LIKE"

      Parameters:
      :   `table` - the table to select from
      :   `column` - the column to select from
      :   `likeColumn` - the x variable for the "where x like y" sql statement
      :   `likeCondition` - the y variable for the "where x like y" sql statement

      Returns:
      :   an array with the selected results


    - #### getSqlSelectByIdByRegExp

      ```
      public static java.lang.String getSqlSelectByIdByRegExp(java.lang.String table,
                                              java.lang.String valueColumn,
                                              java.lang.String idColumn,
                                              java.lang.String id,
                                              java.lang.String regExp)
      ```

      Generates the sql command for a select of a value corresponding to a
      certain id AND matching a certain regex string

      Parameters:
      :   `table` - the table to select from
      :   `valueColumn` - the column to select data from
      :   `idColumn` - the column that contains the id to select by
      :   `id` - the single id to select by
      :   `regExp` - a regular expression pattern (check your database syntax for
          details)

      Returns:
      :   an array with the selected results


    - #### getSqlSelectByRegExp

      ```
      public static java.lang.String getSqlSelectByRegExp(java.lang.String table,
                                          java.lang.String column,
                                          java.lang.String regExp)
      ```

      Generates the sql command for a select matching a certain regex string

      Parameters:
      :   `table` - the table to select from
      :   `column` - the column to select data from
      :   `regExp` - a regular expression pattern (check your database syntax for
          details)

      Returns:
      :   the string with the sql statement text


    - #### getSqlToDropTable

      ```
      public static java.lang.String getSqlToDropTable(java.lang.String tableName)
      ```

      Get sql to drop table

      Returns:
      :   the drop table sql command


    - #### surroundWith

      ```
      public static java.lang.String surroundWith(java.lang.String text,
                                  java.lang.String surrounding)
      ```

      Adds a surrounding string (usually an apex or similar) as prefix and
      suffix of the given text.

      Parameters:
      :   `text` - string to be surrounded
      :   `surrounding` - a string to use as prefix and suffix (for example, an apex)

      Returns:
      :   the text surrounded by the


- Overview
- Package
- Class
- Use
- Tree
- Deprecated
- Index
- Help

- Prev Class
- Next Class

- Frames
- No Frames

- All Classes

- Summary:
- Nested |
- Field |
- Constr |
- Method

- Detail:
- Field |
- Constr |
- Method
